# Friend and foe: β-cell Ca^2+^ signaling and the development of diabetes

**DOI:** 10.1016/j.molmet.2018.12.007

**Published:** 2018-12-24

**Authors:** Paul V. Sabatini, Thilo Speckmann, Francis C. Lynn

**Affiliations:** 1 Diabetes Research Group, BC Children's Hospital Research Institute, Vancouver, British Columbia, Canada; 2 Department of Surgery, University of British Columbia, Vancouver, British Columbia, Canada; 3 Department of Cellular and Physiological Sciences, University of British Columbia, Vancouver, British Columbia, Canada; 4 Department of Internal Medicine, University of Michigan, Ann Arbor, MI, USA

**Keywords:** β-cells, Diabetes, Ca^2+^, CREB, NFAT, Calmodulin, Calcineurin, CaMK

## Abstract

**Background:**

The divalent cation Calcium (Ca^2+^) regulates a wide range of processes in disparate cell types. Within insulin-producing β-cells, increases in cytosolic Ca^2+^ directly stimulate insulin vesicle exocytosis, but also initiate multiple signaling pathways. Mediated through activation of downstream kinases and transcription factors, Ca^2+^-regulated signaling pathways leverage substantial influence on a number of critical cellular processes within the β-cell. Additionally, there is evidence that prolonged activation of these same pathways is detrimental to β-cell health and may contribute to Type 2 Diabetes pathogenesis.

**Scope of review:**

This review aims to briefly highlight canonical Ca^2+^ signaling pathways in β-cells and how β-cells regulate the movement of Ca^2+^ across numerous organelles and microdomains. As a main focus, this review synthesizes experimental data from *in vitro* and *in vivo* models on both the beneficial and detrimental effects of Ca^2+^ signaling pathways for β-cell function and health.

**Major conclusions:**

Acute increases in intracellular Ca^2+^ stimulate a number of signaling cascades, resulting in (de-)phosphorylation events and activation of downstream transcription factors. The short-term stimulation of these Ca^2+^ signaling pathways promotes numerous cellular processes critical to β-cell function, including increased viability, replication, and insulin production and secretion. Conversely, chronic stimulation of Ca^2+^ signaling pathways increases β-cell ER stress and results in the loss of β-cell differentiation status. Together, decades of study demonstrate that Ca^2+^ movement is tightly regulated within the β-cell, which is at least partially due to its dual roles as a potent signaling molecule.

## Graphical abstract

**Figure:**
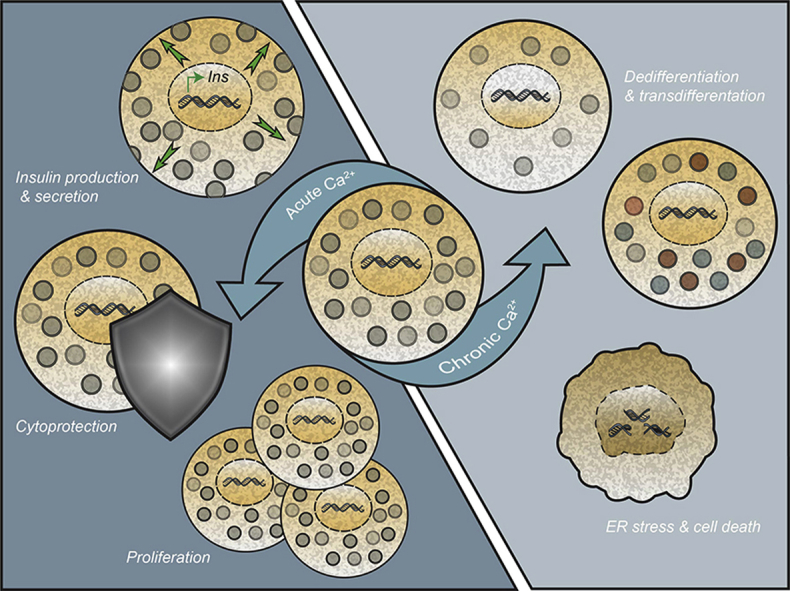

## 1 Introduction

Elevated cytosolic Ca^2+^ (Ca^2+^_i_) initiates a broad range of physiological responses in excitatory cells, from promoting exocytosis in endocrine cells and neurons to muscle contraction in myocytes. These processes are triggered within microseconds of Ca^2+^ influx into the cytosol [1]. Elevations in Ca^2+^_i_ that persist for seconds to minutes produce long-term responses, dependent on the activation of downstream signaling pathways [1]. Dysregulation of the Ca^2+^ signaling cascade contributes to the dysfunction of multiple tissues and cell types in metabolic disorders [2], [3], [4].

Within insulin-producing β-cells, increased Ca^2+^_i_ causes insulin granule exocytosis, but Ca^2+^-mediated signaling pathways also have critical roles in promoting the function, survival, and proliferation of these cells. This review aims to highlight sources of Ca^2+^_i_, important mediators of β-cell Ca^2+^ signaling and their relevance to β-cell biology and type 2 diabetes (T2D).

## 2 Ca^2+^ handling in β-cells

β-cells regulate the systemic response to hyperglycemia through the production and secretion of the hormone insulin. Given the detrimental effects of either impaired or elevated insulin release, the increase in Ca^2+^_i_ that effectively stimulates insulin exocytosis from the β-cell must be closely regulated. This tight control requires the cooperation between multiple Ca^2+^ exchangers, pumps, and channels [5].

In the postprandial state, glucose elicits the influx of Ca^2+^ through L-type voltage-gated Ca^2+^ channels (L-VGCCs). Mediated via glucose metabolism and ATP production, shifts in the ratio of ATP to ADP (ATP:ADP) within the β-cell result in the closure of ATP-sensitive potassium (K_ATP_) channels and membrane depolarization. In human β-cells, L-VGCCs are activated at a membrane potential of −40 mV and, in concert with T-type and P/Q-type Ca^2+^ channels, allow Ca^2+^ influx to elicit insulin exocytosis [6]. Ca^2+^ flux across the β-cell plasma membrane is further regulated by a number of metabolites and nutrients including free fatty acid signaling and cAMP [7], likely through the activation of PKA and subsequent phosphorylation of voltage gated Ca^2+^ channels [8]. Furthermore, hormones including leptin [9] and ghrelin [10] and classical neurotransmitters [11], [12] also regulate Ca^2+^ influx.

**Figure 1:**
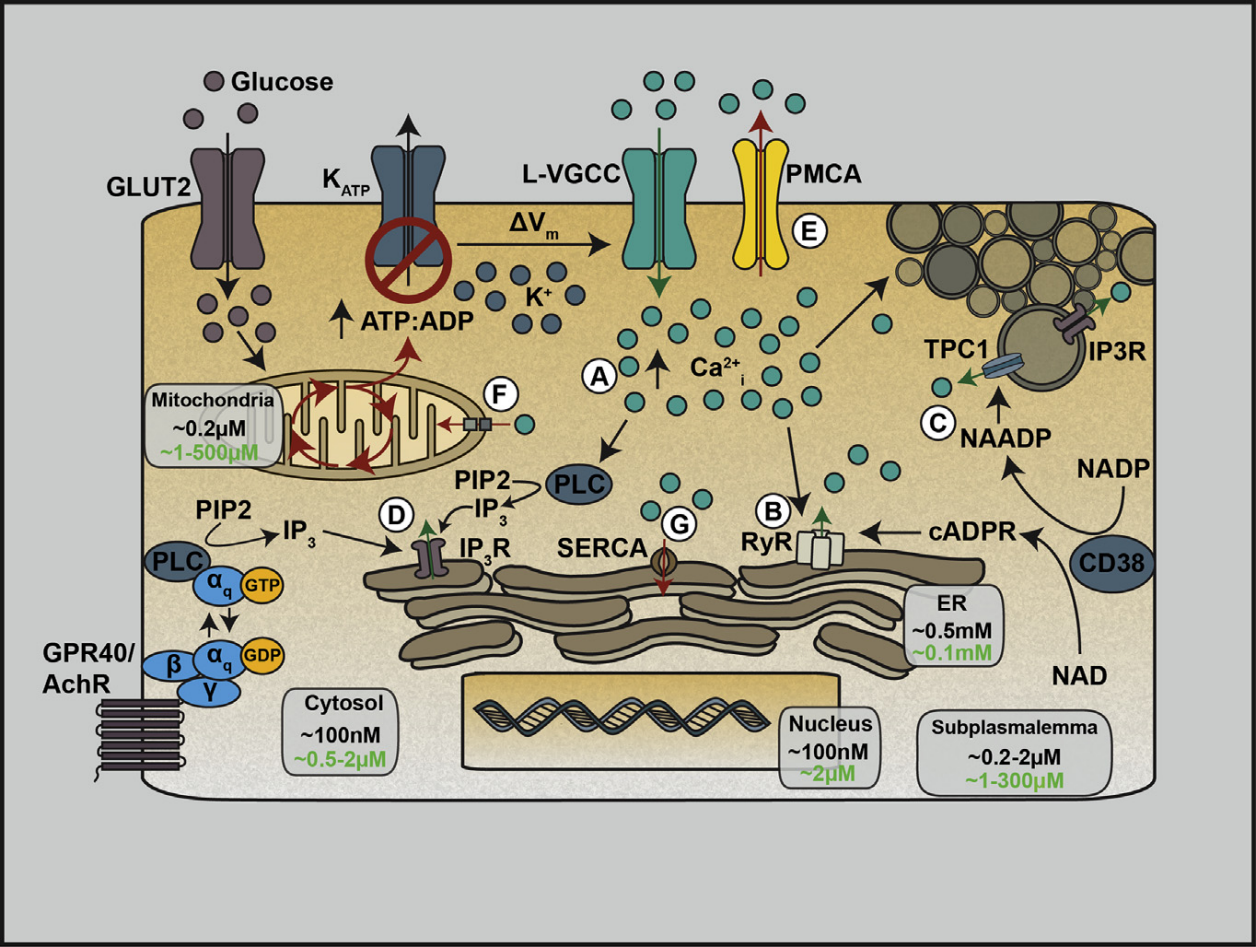
**Schematic of β-cell Ca**^**2+**^**homeostasis pathways**. Extracellular Ca^2+^ influx in β-cells is triggered by the uptake of glucose through glucose transporters (GLUT2 in rodents; GLUT1 in humans) and subsequent metabolism. This shifts the ratio of ATP to ADP, which closes the ATP-sensitive potassium channel (K_ATP_) and opens L-type voltage-gated Ca^2+^ channels (L-VGCCs) (A). There are also intracellular Ca^2+^ pools which contribute to the increase in cytosolic Ca^2+^ (Ca^2+^_i_), including through the ryanodine receptor (RyR) on the ER membrane, through a process termed “Ca^2+^-induced Ca^2+^ release” (B). Additionally, Ca^2+^ is released following glucose metabolism and production of NAADP by CD38, which acts through two pore channel 1 (TPC1) found on acidic vesicles including insulin granules (C). Finally, intracellular Ca^2+^ can be released through the activation of IP_3_ receptors (IP_3_R) found on the ER membrane and on insulin granules. IP_3_Rs are stimulated by the production of IP_3_ from PIP2 following activation of phospholipase C (PLC) by increased Ca^2+^_i_ or by Gα_q_-coupled G-protein receptors including the free fatty acid receptor 1 (FFAR1/GPR40) and acetylcholine receptor (AchR) (D). Following the rise in Ca^2+^_i_ levels, the plasma membrane Ca^2+^ ATPase (PMCA) pumps Ca^2+^ out of the cell (E). Ca^2+^ is also sequestered in the mitochondria by voltage-dependent anion channels and the mitochondrial Ca^2+^ uniporter (F) and the ER through the actions of the sarcoplasmic endoplasmic reticulum Ca^2+^ ATPase (SERCA) (G). Ca^2+^ concentrations within different cellular compartments are shown (black: basal; green: stimulated).

In addition to influx of extracellular Ca^2+^, there are multiple membrane-bound organelles that regulate Ca^2+^_i_ levels, including the nucleus, endoplasmic reticulum (ER), mitochondria, Golgi, as well as vesicles and granules [13], [14]. Intracellular Ca^2+^ stores are distinguished based on their sensitivity to inositol-1,4,5-trisphosphate (IP_3_), nicotinic acid adenine dinucleotide phosphate (NAADP), or ryanodine (summarized in Figure 1). Additionally, intracellular Ca^2+^ stores are responsive to circulating signals, including insulin [15], [16], circulating fatty acids [17], IL-6 [18], and incretin hormones [19], [20], [21], [22], [23].

Ca^2+^ release from IP_3_-sensitive pools occurs through activation of the IP_3_ receptor (IP_3_R), which is expressed on the ER membrane [24], insulin granules, and Golgi [25], [26]. IP_3_ is generated downstream of certain Gα_q_-associated G protein coupled receptors, such as the free fatty acid receptor GPR40 (FFAR1), which signals through phospholipase C (PLC) [27]. PLC then converts phosphatidylinositol-4,5-bisphosphateinositol (PIP2) to IP_3_. Alternatively, PLC is also activated by an increase in Ca^2+^_i_; suggesting other sources of Ca^2+^ (i.e. extracellular, NAADH-responsive) can trigger release of Ca^2+^ from IP_3_R-responsive stores [28], [29].

A second source of Ca^2+^_i_ is the NAADP-responsive pool. Within β-cells, NAADP acts as a second messenger of glucose metabolism, as elevated glucose exposure rapidly increases β-cell NAADP content [30]. NAADP is generated from NADP through ADP-ribosyl cyclases (ARC) such as CD38 [31] and mediates Ca^2+^ release from acidic vesicles such as lysosomes and insulin granules [32] through two pore channel 1 [15], [33], [34]. The NAADP-sensitive stores are required for glucose-stimulated elevations in Ca^2+^_i_, as their inhibition is sufficient to impair glucose-stimulated insulin secretion [35], [36].

The third source for Ca^2+^_i_ is the ryanodine-sensitive pool [37]. The ryanodine receptors (RyRs) are homotetramers with a combined molecular mass of ∼2.3 MDa [38]. While controversy has persisted as to which RyR family members are expressed in β-cells [32], [39], this may be due to the naturally low expression of RyRs, differences in cell type (immortalized cell line or primary tissue) or detection method (less sensitive western blot or PCR), as well as possible differences in splicing. More recently, examination of multiple exons within all three RyR family members in human islets demonstrated detectable expression of all family members [40]. RyRs have been proposed to exist on the β-cell ER [41], insulin granules [32], early endosomes [37] and the plasma membrane [42]. Functionally, RyR channels can be activated by ATP, cAMP and long chain acyl CoA [43], as well as the second messenger cyclic ADP ribose (cADPR), which is produced from NAD^+^ by ARC enzymes, including CD38 [44]. Activation of RyRs promotes glucose-independent insulin release [37]. Additionally, RyRs contribute to glucose-stimulated insulin secretion by mediating the process of Ca^2+^-induced Ca^2+^ release (CICR) [32] through several possible mechanisms, depending on which Ca^2+^ store expresses RyRs. With ER-localized RyRs, CICR is thought to increase Ca^2+^_i_ in close proximity to mitochondria and maintain high rates of ATP generation. Similarly, RyRs expressed on the insulin granule increase Ca^2+^_i_ in the immediate proximity of the insulin granule and facilitate Ca^2+^-dependent vesicle docking and fusion [43].

Beside the regulation of these Ca^2+^-sensitive stores, mitochondria are additional β-cell organelles in which Ca^2+^ handling is tightly regulated and critical for function [45]. Ca^2+^ is exported from mitochondria via the Na^+^/Ca^2+^ exchanger (NCLX) [46], while Ca^2+^ influx into the mitochondrial matrix is achieved through voltage-dependent anion channels (VDACs) in the outer mitochondrial membrane, and the mitochondrial Ca^2+^ uniporter (MCU) complex in the inner mitochondrial membrane [47], [48]. Regulation of MCU by mitochondrial Ca^2+^ uptake 1 (MICU1) is critical for mitochondrial function and β-cell function, as knockdown of MICU1 in INS1 cells reduces Ca^2+^ influx into mitochondria, resulting in reduced glucose-stimulated mitochondrial respiration, ATP production, and insulin secretion [49], [50]. Notably, mitochondrial Ca^2+^ entry from the cytosol is limited by the low affinity of the MCU, but microdomains between the ER and mitochondria (mitochondria-associated membranes; MAMs), tethered through GRP75 and mitofusin 1 and 2, facilitate the rapid transport of large quantities of Ca^2+^ from the ER into mitochondria following IP_3_R- or RyR2-mediated ER Ca^2+^ release [47], [48], [51]. Functionally, Ca^2+^ influx into the mitochondria during periods of high metabolic demands ensures adequate ATP production to maintain insulin secretion by increasing the availability of metabolic substrates and stimulating the TCA cycle (possibly through activation of 2-oxoglutarate dehydrogenase and isocitrate dehydrogenase) [45]. Together, these studies demonstrate the importance of tightly regulated mitochondrial Ca^2+^ levels.

β-cells maintain tight control of Ca^2+^_i_ levels through the regulation of extracellular Ca^2+^ influx and the movement of Ca^2+^ within intracellular depots. The degree of this complexity is illustrated through Ca^2+^ microdomains. Basal levels of free intracellular Ca^2+^ are approximately 100 nM, 20,000× lower than free extracellular Ca^2+^. Following stimulation, whole cell Ca^2+^_i_ increases to 300–1000 nM, but more responsive Ca^2+^ microdomains exist within multiple subcellular locales including dense core vesicles, ER, mitochondria, subplasmalemma, and within the nucleus [52].

Each of these microdomains have functional consequences. The increase in nuclear Ca^2+^ is required for activation of cAMP response element binding (CREB) [53], [54]. Ca^2+^ microdomains surrounding dense core vesicles have been postulated to amplify insulin secretion by increasing Ca^2+^ concentrations in close proximity to Ca^2+^-dependent synaptic proteins [55], and the Ca^2+^ microdomains formed within the mitochondria following high glucose exposure are required for mitochondrial function and second phase insulin secretion [56].

The regulation of Ca^2+^ handling is highly complex, requiring multiple receptors and channels on multiple organelles and the plasma membrane. The potency of Ca^2+^ as a signaling molecule is a major reason for this degree of intricacy.

## 3 Ca^2+^ signaling pathways

Once elevated, Ca^2+^_i_ initiates multiple signaling cascades by binding to and activating the Ca^2+^ sensor protein Calmodulin (CaM). CaM then undergoes a conformational change, allowing it to activate numerous downstream targets [57]. Interaction between CaM and its partners is highly diverse; certain proteins are nearly continuously bound to CaM, while others interact with CaM specifically under either low or high Ca^2+^_i_ conditions [58]. CaM-mediated activation can occur through facilitated dimerization, remodeling of active sites, or removal of autoinhibition [59].

The Ca^2+^/Calmodulin-dependent protein kinases (CaMK) are one class of proteins activated by Ca^2+^-bound CaM. Of the CaMK isoforms [60], CaMKK1 [61], CaMKK2 [62], traces of CaMKI isoforms (α, γ, δ) [63], [64], [65], all CaMKII isoforms (α, β, γ, δ) [66], and CaMKIV [61] have been detected in β-cells. Targets of the CaMKs include the transcription factor CREB. Under low Ca^2+^ conditions, inactive CREB is bound to consensus sites (TGACGTCA) [67], whereas increases in Ca^2+^_i_ result in CREB activation through a CaMK-dependent pathway [68], [69]. Phosphorylated CREB then interacts with its co-factors CREB regulated transcription coactivator 2 (CRTC2) and CREB binding protein (CBP) to promote target gene transcription [70]. Besides Ca^2+^_i_-mediated phosphorylation of CREB, Ca^2+^ signaling pathways also increase CREB activity via CRTC2. Activation of the phosphatase Calcineurin (CaN) results in the dephosphorylation of cytoplasmic CRTC2, which subsequently dissociates from cytoplasmic 14-3-3 chaperone proteins and translocates to the nucleus, where it increases CREB transcriptional activity [71]. CRTC2 is exported from the nucleus following re-phosphorylation by microtubule affinity regulating kinase 2 (MARK2) [72] and salt inducible kinase 2 (SIK2) [71].

Independent of the CaMK/CREB pathway, CaM also activates a separate signaling cascade through CaN. CaN has many target proteins, including nuclear factor of activated T cells (NFAT) [73] and myocyte enhancer factor-2 (MEF2) [74] family members. CaN-mediated dephosphorylation results in NFAT nuclear translocation and transcriptional activation [73]. NFAT proteins are exported from the nucleus via re-phosphorylation by the kinases DYRK1A and GSK3β [75].

**Figure 2:**
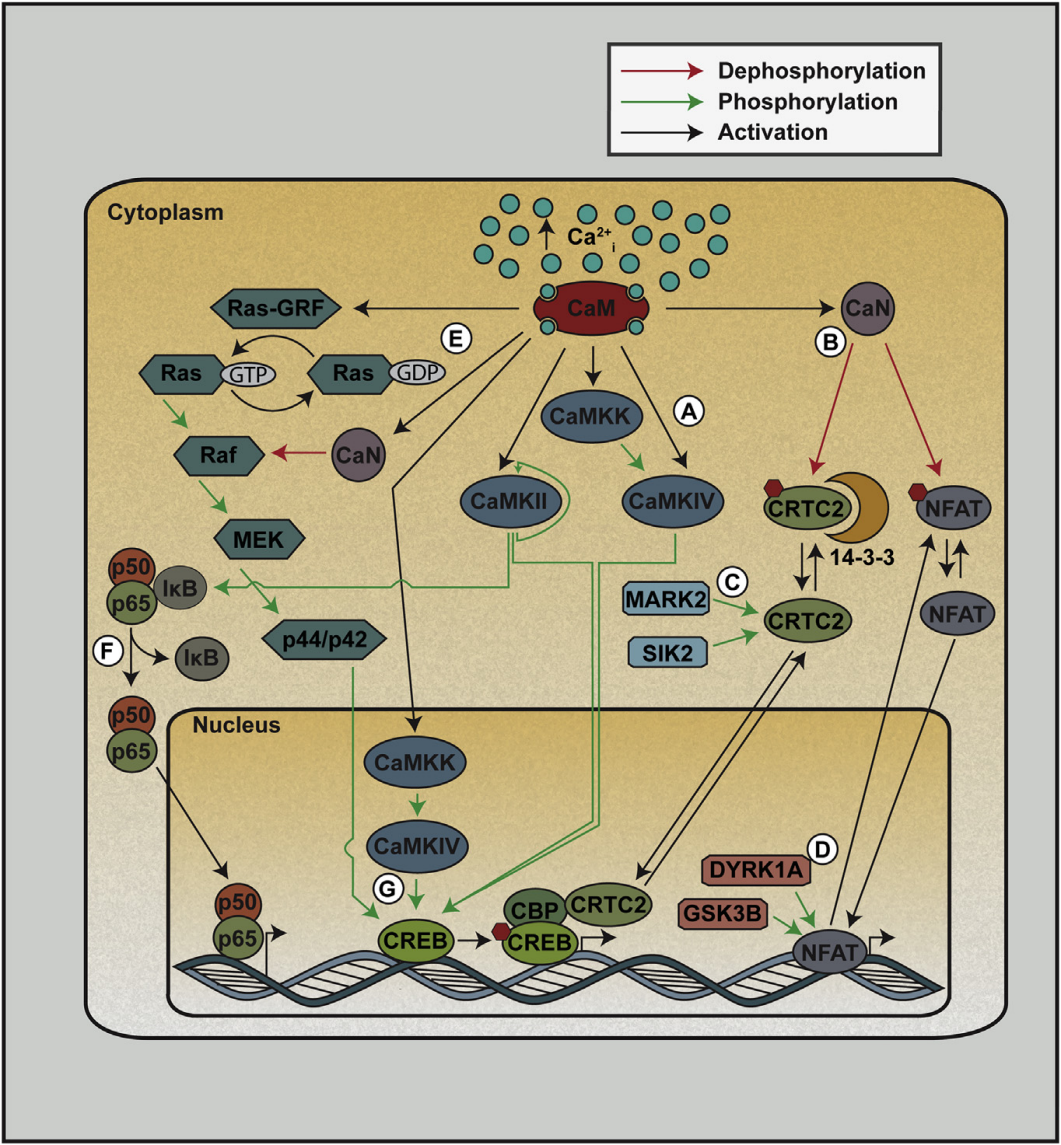
**Ca**^**2+**^**signaling pathways in β-cells**. Following Ca^2+^ binding to Calmodulin (CaM), multiple downstream pathways are activated. CaM activates the Ca^2+^/Calmodulin-dependent protein kinase kinase (CaMKK) as well as members of the Ca^2+^/Calmodulin-dependent protein kinases (CaMK). Ca^2+^/CaM-bound CaMKK can phosphorylate and activate CaMKIV (A). Ca^2+^-bound CaM also activates the phosphatase Calcineurin (CaN) (B), which removes phosphate groups from CREB regulated transcription coactivator 2 (CRTC2) and nuclear factor of activated T cells (NFAT) proteins, resulting in their nuclear localization. CRTC2 can be inactivated through phosphorylation by microtubule affinity regulating kinase 2 (MARK2) and salt inducible kinase 2 (SIK2) (C), and NFAT can be inactivated by the kinases GSK3B and DYRK1A (D). Ca^2+^ results in the activation of p44/p42 (ERK1/2) or the MAP kinase pathway through the stimulation of Ras-GRF by CaM and the dephosphorylation of Raf by CaN (E). The NF-κB pathway can also be activated by Ca^2+^ in β-cells through the phosphorylation of IκB, which releases the p50 and p65 subunits (F). The upstream activation of various CaMKs and members of the MAP kinase pathway result in the phosphorylation and activation of cAMP response element binding (CREB) (G).

In addition to CaMK and CaN pathways, increased Ca^2+^_i_ in β-cells activates other proteins and signaling cascades, including the MAP kinase pathway. This is mediated through the activation of Ras-GEF and B-Raf via CaM [76] and CaN [77], [78], [79], respectively, and results in activation of p42/44 (ERK1/2) [80]. Additionally, both p38 MAPK [81] and NF-κB [82] are activated by elevated Ca^2+^_i_ in β-cells. CaMKII mediates the activation of NF-κB activation in β-cells through the phosphorylation of IκBα [82], a known target of CaMKII in neurons [83] (Figure 2). The temporal dynamics and sensitivity to Ca^2+^_i_ of each of these pathways are not well defined in β-cells. While computational modeling predicts that increasing frequency of Ca^2+^_i_ oscillations preferentially activates CaMKII over CaN [84], experimental data generated in β-cells are needed.

The disparate pathways active by elevated Ca^2+^_i_ suggest that Ca^2+^ is a central mediator of many different cellular processes within the β-cell. Indeed, the study of the mediators and effectors of these Ca^2+^ signaling pathways demonstrates their importance in maintaining β-cell function and glucose homeostasis.

## 4 The role of Ca^2+^ in insulin production and secretion

During periods of elevated metabolic demand, β-cells must increase the production of insulin to ensure adequate insulin stores are maintained. As such, high glucose exposure increases insulin production in rat islets [85]. Influx of extracellular Ca^2+^ is critical for this process, as blocking L-VGCCs with verapamil ameliorates glucose-mediated insulin transcription [86]. The Ca^2+^-mediated promotion of insulin transcription can be separated into NFAT- and CaMK-dependent pathways. The rat insulin 1 promoter contains multiple NFAT binding sites [87], and NFATC2 is enriched at the insulin promoter following high glucose exposure in MIN6 cells and human islets [88]. Please note, immortalized β-cell lines have abnormal rates of apoptosis and replication and likely have abnormal activation of Ca^2+^ signaling pathways; therefore, conclusions derived from cell lines should be verified in primary cells. Furthermore, inhibiting NFAT with the CaN inhibitor tacrolimus (FK-506) abrogates the glucose-mediated increase in insulin promoter activity in INS-1 cells [87]. NFAT proteins are also sufficient to increase insulin gene expression, since a β-cell specific doxycycline-responsive constitutively active NFATC2 significantly increases *Ins1* and *Ins2* gene expression *in vivo* [89].

Besides NFAT, CaMKIV is also required to induce insulin expression, as shown by transfection of INS-1 cells with a kinase-dead CaMKIV, which blocks glucose-mediated elevations in insulin promoter activity [61]. Conversely, overexpression of constitutively active CaMKIV significantly increases insulin gene expression in INS-1 cells [61]. CaMKIV may promote insulin expression through the actions of the transcription factors ATF2 (CREB2) and EGR1. Both ATF2 and EGR1 are positively regulated by Ca^2+^ in CaMKIV- and SRF-dependent manners, respectively, and overexpression of either factor is sufficient to increase insulin promoter activity [90], [91], [92]. The promotion of insulin production downstream of NFAT and CaMK pathway activation creates a system wherein Ca^2+^, acting as a stimulus for insulin secretion and also a signal to increase insulin synthesis, ensures adequate insulin levels during prolonged stimulation. In addition to the transcriptional regulation of insulin by members of Ca^2+^ signaling pathways, elevated glucose also increases rates of insulin mRNA translation [93] and stabilizes insulin mRNA [94]. However, the role of Ca^2+^ signaling pathway members in these processes is unknown.

While increases in Ca^2+^_i_ are required for insulin granule fusion to the plasma membrane, activation of Ca^2+^ signaling pathways also promotes insulin secretion through CaMK- and CaN-dependent pathways. The importance of Ca^2+^ signaling pathways in promoting insulin secretion is observed in mouse models wherein diminished activity or expression of CaMKII [95], CREB [96], CaM [97] or CRTC2 [98] in mouse β-cells impairs insulin secretion and systemic glucose homeostasis. Furthermore, pharmacological inhibition of CaN with either FK-506 or cyclosporin A decreases insulin secretion in human islets [99], [100], while overexpression of NFATC1 and NFATC2 increases glucose- and KCl-stimulated insulin secretion in mouse islets [101]. These *in vitro* and *in vivo* models all support a critical role for members of Ca^2+^ signaling pathways in the promotion of insulin secretion.

One mechanism through which Ca^2+^ signaling promotes insulin secretion is through the formation β-cell “metabolic memory”, wherein repeated exposure to elevated glucose primes β-cells to significantly increase insulin secretion during an ensuing high glucose exposure [102]. Inhibiting CaMKII activity with KN93 abrogates the augmentation of insulin secretion during the secondary glucose challenge, suggesting a critical role for this kinase in the formation of a metabolic memory [102]. While the precise mediators which form the β-cell metabolic memory have not been elucidated, repeated high glucose exposure increases the expression of glucokinase, SNAP25, and MAFA. Additionally, phosphorylation levels of Synapsin I, a direct target of CaMKII, are increased following repeated high glucose exposure [103].

Ca^2+^ signaling may also promote insulin secretion by elevating mitochondrial activity through a process termed “Ca^2+^-metabolic coupling”. Periods of elevated insulin secretion require increased mitochondrial activity to replenish the ATP stores that sustain ATP-mediated membrane depolarization and insulin release. Influx of Ca^2+^ and downstream activation of CaMKs is required for this prolonged elevation in mitochondrial function, as inhibiting L-VGCCs or CaMKs blocks increased oxygen consumption rate (OCR; a measure of mitochondrial function) [104], [105], [106]. Furthermore, directly stimulating L-VGCCs with BayK8644 increases β-cell OCR, demonstrating the tight coupling of Ca^2+^_i_ with mitochondrial function [105].

These studies establish that, in addition to Ca^2+^-mediated insulin vesicle fusion, activation of CaN/NFAT and CaMK also promote insulin secretion by increasing mitochondrial respiration and priming the β-cell under repeated high glucose exposures.

## 5 The role of Ca^2+^ in β-cell replication

Increased rates of β-cell proliferation are one adaptive mechanism β-cells employ to compensate for elevated metabolic demand and ensure euglycemia is maintained. Both *in vitro* [107] and *in vivo* studies [108], [109] have observed that increased β-cell proliferation in response to elevated glucose concentrations and Ca^2+^ signaling is critical for this process. Pharmacologic stimulation of glucokinase also increases β-cell replication [110], [111], which can be blocked by inhibiting membrane depolarization with diazoxide [110], suggesting that Ca^2+^ influx, as opposed to glucose metabolism alone, is necessary. Furthermore, increasing Ca^2+^_i_ with the L-VGCC agonist, BayK8644, induces rat β-cell proliferation [112], [113], providing additional support for the role of Ca^2+^ signaling pathways in promoting β-cell proliferation.

Both CaMK- and NFAT-dependent mechanisms mediate the mitogenic effects of elevated Ca^2+^_i_ in β-cells. Blocking CaMK activity with KN62 abrogates the glucose-mediated increase in β-cell proliferation [114]. Additionally, overexpression of constitutively active CaMKIV or dominant-negative CaMKIV significantly elevates or diminishes β-cell proliferative rates, respectively [114]. Downstream of CaMKIV, CREB activity is also required, as co-expression of a dominant-negative CREB can abrogate the mitogenic effects of CaMKIV overexpression and the CREB targets *Irs2* and *Nr4a1* promote β-cell proliferation [69], [107], [114], [115], [116], [117]. In sum, these data suggest that the CaMKIV/CREB/*Irs2* and *Nr4a1* pathway is one mechanism by which elevations in Ca^2+^_i_ promote β-cell replication.

NFAT proteins also promote β-cell replication. Islets from juveniles (age 0.5 to nine years old) have higher proliferation rates associated with higher expression of *NFATC1*, *NFATC2*, and *NFATC4* than islets from adults (20 years or older) [118]. Additionally, the expression of a doxycycline-mediated constitutively nuclear NFATC2 in mice increases β-cell proliferation rates 2-fold *in vivo* [89]. Within cultured human islets, overexpression of constitutively active NFATC1 or NFATC2 increases proliferation rates by 2- and 3-fold, respectively [101]. In support of the proliferative role of NFAT proteins in β-cells, two unbiased small molecule screens identified β-cell mitogens that act by inhibiting the NFAT kinases DYRK1A and GSK3β, thus increasing NFAT activity [112], [119]. These small molecule screens have been validated by an independent study, which found that the small molecule 5-iodotubercidin inhibits multiple DYRK family members and induces human β-cell proliferation through a CaN-dependent pathway [120]. Finally, increases in CaN activity may mediate the proliferative effects of the GLP-1 receptor agonist, exendin-4, on β-cells. Exendin-4-treated human islets have a 2-fold increase in proliferation rates and an associated significant increase in *NFATC1*, *NFATC3*, and *NFATC4* expression. Inhibition of CaN with FK-506 abrogated exendin-4-mediated increases in NFAT gene expression level and β-cell proliferation rates [118]. Mechanistically, NFAT proteins transcriptionally regulate a large number of cell cycle and mitogenic genes in β-cells [101], including direct induction of *Irs2* [121], [122], *Ccdn1*, and *Cdk4* [89], which may all promote β-cell proliferation.

Similar to the positive effect of Ca^2+^ signaling pathways on insulin production, elevated β-cell proliferation rates during periods of increased systemic insulin demand allow for appropriate β-cell compensation and ensure appropriate β-cell functional capacity to maintain euglycemia.

## 6 The role of Ca^2+^ in β-cell survival

Ca^2+^ signaling pathways also promote β-cell viability and survival. MIN6 cells incubated for 24 h in high glucose (25 mM) have significantly reduced rates of apoptosis compared to MIN6 incubated in low glucose (5 mM) concentrations [123]. The cytoprotective effects of elevated glucose are blocked by inhibiting depolarization with diazoxide or Ca^2+^ influx with nifedipine [123]. Both CaN- and CaMK-dependent pathways have been suggested to mediate the pro-survival effects of Ca^2+^.

Inhibition of CaN with either FK-506 or cyclosporine A induces β-cell apoptosis in human islets *in vitro* [100], and FK-506 treatment of diabetic mice transplanted with human islets impairs graft function and glucose homeostasis [100], [124]. Examination of pancreatic biopsies from individuals receiving either cyclosporine A or FK-506 as an immunosuppressant display cellular evidence of β-cell apoptosis [125]. Finally, use of CaN inhibitors FK-506, cyclosporine A, and sirolimus as immunosuppressants in solid organ transplant is associated with the development of impaired glucose homeostasis and diabetes [126], [127]. These results suggest that CaN activity is required for β-cell survival.

In addition to the role of NFAT proteins, the CaMKIV/CREB pathway also promotes β-cell viability. MIN6 cells incubated in 12.5 mM glucose have significantly reduced caspase-3 activity compared to MIN6 incubated in 2.5 mM glucose [114]. CaMKIV may mediate these effects, as expression of a constitutively active CaMKIV reduces β-cell apoptosis rates and co-expression of a dominant-negative CREB is sufficient to block the cytoprotective effects of increased CaMKIV activity [114]. Supporting the role of CREB in promoting β-cell viability, *in vivo* studies show that transgenic expression of a dominant-negative CREB (A-CREB) in β-cells increases apoptosis and results in diabetes in mice [68], and *in vitro* studies demonstrate knockdown of CREB in INS-1 cells increased levels of cleaved caspase-3 [128]. CREB may promote β-cell viability through induction of cytoprotective factors *Irs2* and *Npas4*, which both protect β-cells from stress and cell death [68], [129], [130].

In addition to the ability of elevated Ca^2+^_i_ to promote β-cell viability, decreased Ca^2+^_i_ also adversely impacts β-cell survival by impairing ER and mitochondrial Ca^2+^ handling. Depletion of ER Ca^2+^ results in ER stress and β-cell apoptosis [131], [132], [133]. Mechanistically, during states of low β-cell Ca^2+^_i_, such as low glucose exposure, ER Ca^2+^ depletion occurs due to inactivation of the sarcoplasmic endoplasmic reticulum Ca^2+^ ATPase (SERCA) and the ensuing lack of ER Ca^2+^ uptake [134]. Ca^2+^_i_ is also intricately connected to mitochondrial function, as the activity of several mitochondrial enzymes depends on Ca^2+^ [135]. Mitochondrial Ca^2+^ uptake follows depolarization-dependent increases in Ca^2+^_i_, and ATP production increases as a consequence [136], [137]. Thus, decreases in Ca^2+^_i_ are predicted to decrease mitochondrial activity, ATP production and SERCA action; and in so doing promote ER Ca^2+^ depletion, ER stress and β-cell death.

## 7 Ca^2+^ signaling pathways in T2D

As outlined above, Ca^2+^ signaling pathways have critical roles in regulating β-cell function, proliferation and viability; all processes that fail during the development of T2D. Despite this importance, only few members of Ca^2+^ signaling pathways have appeared as susceptibility loci in T2D GWAS studies [138], including the likely causal *CDC123/CAMK1D* locus [139], [140] and *CAMKK2* variants [141]. However, a recent analysis of regulatory elements upstream of T2D susceptibility genes identified *NFATC2* as a regulatory factor for 40% of genes identified through GWAS [101]. Furthermore, overexpression of NFATC1 or NFATC2 in human islets significantly alters the expression of a number of T2D susceptibility genes including *KLF11*, *HHEX*, and *PROX1* [101]. While the genetic link between Ca^2+^ signaling pathways and T2D requires further examination, research using animal models and clinical data support a role for impaired Ca^2+^ signaling in β-cell failure during T2D pathogenesis.

The prediabetic milieu, characterized by increased glucose and fatty acids levels, results in increased β-cell depolarization, Ca^2+^ influx, and insulin secretion to maintain euglycemia. Short-term stimulation of Ca^2+^ signaling pathways yields positive effects for the β-cell (insulin production, secretion, replication and viability). In contrast, chronic stimulation of these pathways has deleterious effects. This is observed in multiple rodent models in which members of Ca^2+^ signaling pathways are overexpressed. For instance, overexpression of a constitutively active CaN increases β-cell apoptosis, decreases proliferation, and results in glucose intolerance [142]. Similarly, mice that overexpress a constitutively active CaMKIIα in β-cells also develop diabetes associated with decreased β-cell mass [143]. CaM overexpression in mouse β-cells also leads to diabetes [144]. In this CaM overexpression model, there is also a loss of insulin-expressing cells with a concomitant increase in islet cells expressing glucagon, perhaps due to β-cell transdifferentiation into α-cells [144]. The observations from mouse models suggest chronic activation of Ca^2+^ signaling pathways impairs β-cell function, which is supported by human studies in which individuals with T2D are treated with diazoxide to inhibit β-cell depolarization. After a multi-day treatment period, insulin secretion is improved [145], [146]. This model of pathogenic Ca^2+^ flux may also explain why sulphonylureas initially improve, but ultimately worsen, glycemic control in individuals with T2D [147].

Chronically elevated Ca^2+^_i_ may drive β-cell dysfunction and failure by exacerbating ER stress and β-cell differentiation. β-cell ER stress is sufficient to cause diabetes in mice [148], has been observed in individuals with T2D [149], and relies on activation of Ca^2+^ signaling pathways [4]. Treatment with a combination of high glucose and palmitate results in stark activation of ER stress and increases rates of β-cell apoptosis in both immortalized β-cell lines and primary islets [150], [151]. However, blocking depolarization with diazoxide [150] or Ca^2+^ influx with nifedipine [151] protects against the induction of ER stress and subsequent apoptosis of β-cells.

In addition to exacerbation of ER stress, chronically active Ca^2+^ signaling also results in loss of β-cell maturation. Models in which β-cells are exposed to chronically elevated glucose levels and increased Ca^2+^_i_ result in the loss of β-cell maturation; such as the *db/db* or Akita mouse, a diphtheria toxin-mediated β-cell ablation model, insulin receptor antagonism [152], or genetic removal of insulin genes from β-cells [153]. Furthermore, inhibiting β-cell depolarization in the *db/db* mouse, by crossing it to a β-cell specific constitutively active *Kir6*.*2* mutant, significantly reduces rates of β-cell transdifferentiation into gastrin-expressing cells compared to *db/db* controls, despite no improvement in glucose handling [152]. This experiment strongly suggests that β-cell depolarization, and not hyperglycemia alone, is required to drive β-cell dedifferentiation. This hypothesis is supported by an *in vitro* transdifferentiation model in which mouse islets are cultured at either 5 mM or 25 mM glucose. After 2 days in culture, islets exposed to high glucose have a significant increase in gastrin expression, which could be abrogated by co-culture with either diazoxide, nifedipine or FK-506 [152], demonstrating that Ca^2+^ influx and CaN activity are also required for this process. Importantly, islets from individuals with T2D have significantly increased numbers of gastrin-expressing cells; although it remains unknown whether aberrant Ca^2+^ signaling is the cause [152].

The role of Ca^2+^ signaling in driving β-cell transdifferentiation is further supported by data from the β-cell *Abcc8* knockout mouse. Deletion of *Abcc8*, a subunit of the K_ATP_ channel, from β-cells increases intracellular Ca^2+^ most notably under low glucose conditions, but also under high glucose exposure [154]. This is accompanied by the loss of β-cell maturation status and transdifferentiation into PP-cells, despite an absence of frank hyperglycemia [154]. Additionally, expression of the dedifferentiation marker *Aldh1a3* is significantly increased in the *Abcc8* null mouse and can be largely normalized by blocking Ca^2+^ influx with verapamil [154]. In contrast to other dedifferentiation models, which present with profound hyperglycemia, this *Abcc8* null model decouples hyperglycemia from increased Ca^2+^ influx and elegantly demonstrates that chronically active Ca^2+^ signaling pathways are sufficient to promote β-cell dedifferentiation.

These studies define a clear role for Ca^2+^ signaling pathways in driving β-cell dedifferentiation and transdifferentiation and support a model in which chronic activation of Ca^2+^ signaling pathways results in increased stress and a loss of β-cell maturation status that contributes to β-cell failure in T2D. Less certain, however, is the role of altered Ca^2+^ handling in the development of type 1 diabetes (T1D). *In vitro* models of cytokine treatment show impaired β-cell Ca^2+^ handling following exposure to proinflammatory cytokines, including reduced oscillatory Ca^2+^ fluctuations [155] and impaired glucose-stimulated Ca^2+^ influx [156]. Additionally, blockage of L-VGCCs protects mice from diabetes and β-cell loss in low-dose STZ-induced diabetes [157]. However, further assessments in T1D models such as the non-obese diabetic mouse may bring further illumination as to the role of Ca^2+^-regulated cell death in T1D.

## 8 Conclusions and future directions

In the postprandial state, β-cells undergo waves of depolarization and Ca^2+^ influx, which activates multiple downstream signaling pathways. Stimulation of these pathways promotes insulin production and secretion, proliferation, and viability. The importance of Ca^2+^ signaling in β-cells is evidenced by the β-cell dysfunction and impairment in systemic glucose homeostasis that results from inhibiting the activity of various members of the Ca^2+^ signaling cascade, including CREB [68], [96], CaN [89], and CaMKII [95]. Conversely, overstimulation of these pathways (a summary of mouse models in Table 1), as is observed under chronic hyperglycemia, also results in β-cell dysfunction and loss of β-cell differentiation status. This is in line with observations from mouse models that specifically overexpress CaN [142], CaMKII [143], or CaM [144], [158], which impairs β-cell function, maturation status, and viability. Together, these studies underscore the requirement for tight control over Ca^2+^_i_ and the downstream pathways it regulates in β-cells.

**Table 1:** Mouse models of Ca^2+^-related diabetes.

Target gene	Model	Phenotype	Reference(s)
**Models of decreased expression/activity**			
*Calm1*	Transgenic OE of inactive *Calm1* (CaM-8)	Reduced insulin secretion resulting in diabetes	[97] Ribar et al., 1995
*Camk2* (*a*,*b*,*d*,*g*)	Tetracycline-mediated OE of *Camk2* pseudosubstrate inhibitory peptide (EAC3I)	Reduced insulin secretion and impaired glucose tolerance	[95] Dadi et al., 2014
*Creb1*	Transgenic OE of DN *Creb1* (A-CREB)	Increased apoptosis resulting in diabetes	[68] Jhala et al., 2003
	Pdx1-CreER^Tg^-mediated deletion	Females on HFD glucose intolerant (not observed in males)	[96] Shin et al., 2014
*Crtc2*	MIP-CreER-mediated deletion	Reduced insulin secretion and glucose intolerance	[98] Blanchet et al., 2015
*Ppp3r1*	Ins2-Cre^Tg^-mediated deletion of calcineurin b1	Diabetes after 10 weeks	[89] Heit et al., 2006
*Nr4a1*	Germline deletion	Reduced β-cell proliferation	[117] Tessem et al., 2014
*Abcc8*	Ins2-Cre^Tg^-mediated deletion	β-cell transdifferentation into PP-cells	[154] Stancill et al., 2017
**Models of increased expression/activity**			
*Calm1*	Transgenic OE of chicken *Calm1* in β-cells	Increased apoptosis, possible transdifferentiation resulting in diabetes	[144] Epstein et al., 1989
			[158] Yu et al., 2002
*Camk2a*	Transgenic OE in β-cells	Increased apoptosis, decreased proliferation resulting in diabetes	[143] Kato et al., 2008
*Nfatc1*	Doxycycline-inducible transgenic OE of constitutively active *Nfatc1* (Nfatc1^nuc^)	Increased Pdx1, Glut2, and β-cell proliferation	[89] Heit et al., 2006
*Ppp3ca*	Transgenic OE of constitutively active calcineurin A in β-cells	Reduced proliferation/increased apoptosis resulting in diabetes	[142] Bernal-Mizrachi et al., 2010

Summary of mouse models of diabetes or β-cell dysfunction following overexpression (OE) or deletion of Ca^2+^-related genes. DN = dominant-negative; HFD = high fat diet; Tg = transgenic.

While current studies have observed disrupted β-cell Ca^2+^ handling in mouse models [159], [160] and in humans with T2D [125], [126], [127], [161], there are several avenues of research which offer greater understanding of the pathogenic role of altered Ca^2+^ signaling in the β-cell. These include a further characterization of how altered Ca^2+^ signaling impacts β-cell transcriptomics, ER and mitochondrial function, and defining the nature of the altered Ca^2+^ handling by the β-cell under pathologic conditions, particularly by important Ca^2+^ stores such as ER and mitochondria. Finally, it will be important to determine if and how Ca^2+^ signaling pathways are impaired in β-cells from individuals with T2D and whether these pathways can be therapeutically targeted.
